# Self-Compassion, Work Engagement and Job Performance among Intensive Care Nurses during COVID-19 Pandemic: The Mediation Role of Mental Health and the Moderating Role of Gender

**DOI:** 10.3390/healthcare11131884

**Published:** 2023-06-29

**Authors:** Reihaneh Bahrami Nejad Joneghani, Rayehe Bahrami Nejad Joneghani, Hakimeh Dustmohammadloo, Parisa Bouzari, Pejman Ebrahimi, Mária Fekete-Farkas

**Affiliations:** 1 Industries Engineering (System and Utilization), Islamic Azad University-Najafabad Branch, Isfahan 85141-43131, Iran; rahabahrami@ymail.com; 2 Business Management (Marketing), University of Isfahan, Isfahan 85141-43131, Iran; rayehebahrami67@gmail.com; 3 California Language Academy, Jalan Ampang, Kuala Lampur City Center, Kuala Lampur 50450, Malaysia; dostmohamadloo888@gmail.com; 4 Department of Agricultural Logistics, Trade and Marketing, Institute of Agricultural and Food Economics, Hungarian University of Agriculture and Life Sciences (MATE), Páter Károly Street 1, H-2100 Gödöllő, Hungary; bouzari.parisa@stud.uni-mate.hu; 5 Doctoral School of Economic and Regional Sciences, Hungarian University of Agriculture and Life Sciences (MATE), H-2100 Gödöllő, Hungary; 6 Institute of Agricultural and Food Economics, Hungarian University of Agriculture and Life Sciences (MATE), Páter Károly Street 1, H-2100 Gödöllő, Hungary; farkasne.fekete.maria@uni-mate.hu

**Keywords:** COVID-19 pandemic, job performance, mental health, self-compassion, work engagement

## Abstract

(1) Background: The COVID-19 pandemic has highlighted attention on the importance of certain variables in predicting job performance. Among these, mental health is one of the main variables affected by this pandemic. It can have an important mediating role in predicting job performance by individual, occupational, and organizational variables, especially in the nursing community. However, there is little information about its mediation role among the predictors of job performance. This cross-sectional study aimed to examine the role of mental health as a mediating factor in the influence of self-compassion and work engagement on ICU nurses’ job performance during the COVID-19 pandemic, as well as the moderating effect of gender on all model relations. A survey of 424 ICU nurses (men 193 and women 231) was undertaken in three Coronavirus hospitals in Isfahan, Iran. (2) Method: Questionnaires were distributed and collected among the statistical sample, and the data from the questionnaires were analyzed using AMOS24 software (version 24). The research model was evaluated in two stages (the main model and the two sub-models in two gender groups). (3) Result: The analysis revealed that work engagement (β = 0.42, *p* < 0.001), mental health (β = 0.54, *p* < 0.001) and job performance (β = 0.51, *p* < 0.001) were discovered to be positively related to self-compassion. Work engagement is positively associated with mental health (β = 0.16, *p* < 0.01) and job performance (β = 0.21, *p* < 0.001), and mental health is linked positively to job performance (β = 0.23, *p* < 0.001). Furthermore, the effects of self-compassion and work engagement on job performance are mediated by mental health. According to the findings, gender moderates the link between self-compassion and work engagement, work engagement and job performance, and self-compassion and job performance. (4) Conclusion: Mental health has a mediating role in the effect of self-compassion and work engagement on ICU nurses’ job performance. Gender also acted as a moderator in some relationships. Males are dominant in all of these relationships as compared to females.

## 1. Introduction

Following the prevalence of COVID-19 in China in 2019 and the confirmation of its pandemic, the pandemic became an international concern in January 2020 [1]. Nurses’ mental health was at risk during this crisis due to the high workload and stress they endured. The increasing trend in cases and deaths associated with the coronavirus, fear of transmission of the virus to the family, increased workload, lack of equipment, shortages and, in some cases, lack of specific drugs or appropriate treatment are factors affecting the occurrence of mental health issues in this group [2,3]. In acute cases, these problems have even led to suicides among nurses in countries such as India and Italy [4,5], which shows the importance of this structure. In addition, this group’s continuous mental health problems can have devastating effects on the process and performance of their professional activities [6,7]. In Iran, COVID-19 has also become widespread, causing mental health problems for healthcare professionals [8].

Therefore, recognizing the factors affecting nurses’ mental health to intervene and improve their results can be especially important in this period when the intensity of tension and stress is much higher than usual. In addition, mental health may also act as a mediator in predicting job performance through antecedent variables [9]; this highlights the need for further research on the effects of this variable.

Self-compassion is one of the newest approaches as a topic of empirical interest among researchers [7,10], and it has appeared as a structure concerned with employee mental health and well-being in both organizations and hospitals [11,12,13,14,15,16]. The explanation of such relationships can be explained by Gilbert’s regulatory model [17] and Key Resources Theory (KRT) [18,19]. However, there have been studies undertaken on self-compassion and its adverse impacts, such as avoidance and anxiety attachment, and its positive impacts, such as stress management, work performance, organizational social behaviors and job retention [10,15,20]. Research into this structure and its connections with critical processes and outcomes is still in the initial phase.

Another important factor that has been proposed to promote mental health and job performance and has been mentioned in related research to the structure of compassion is the work engagement of employees. However, the relationship between these variables needs further study. Work engagement is a structure that has been considered in organizational studies and in the field of hospitals to maintain nurses’ high job performance in stressful situations [21]. According to Kahn [22], work engagement is a motivational concept that gives workers vigor and enthusiasm to perform their work tasks [23]; therefore, it leads to higher job performance and greater employee satisfaction [24]. This variable requires motivational resources that are related to employees’ mental health at the workplace. Therefore, high work engagement means having work motivation, and in previous research, it has been linked to fewer mental health problems and less mental health shame among staff and business students [25,26].

Another remarkable point is that some research has looked into and confirmed the impact of compassion on workplace engagement [27,28]. However, there are only a few scattered studies in relation to self-compassion and its effect on work engagement [26,29], whose possible impact can be based on the Self-Determination Theory (SDT) [26,30]. In this context, it seems that the research has not specifically examined the relationship between self-compassion and work engagement, and there are no accurate results. Therefore, examining this relationship in the present research can help expand the theoretical foundations in this field. Based on the mentioned relationships, in addition to examining the direct effect of self-compassion and job engagement on job performance, the present study also investigates the mediating role of mental health. This has not been studied so far.

According to social role theory (SRT), it is important to explore psychological behaviors and expectations, which can be different between men and women under the influence of social norms [19,31]; therefore, it is essential to investigate gender differences.

This study aimed to investigate the effects of self-compassion and work engagement on job performance through mental health and the moderating role of gender among nurses in the ICU of three coronavirus hospitals in Isfahan, Iran, within a cross-sectional study.

## 2. Theoretical Research Framework

### 2.1. Effect of Self-Compassion on Mental Health and Job Performance

Self-compassion is a generalized variable of compassion but is also different. Self-compassion focuses on self-care that, with awareness and acceptance of all aspects of ourselves, promotes resilience and perseverance in difficult times and stressful situations [10,32].

It consists of three interconnected components: self-kindness versus critical self-judgment, common humanity versus isolation, and mindfulness versus over-identification [33].

In addition to the explanations based on Gilbert’s model, which was stated in the introduction, self-compassion includes kindness, intimacy, and a sense of reciprocity; these characteristics assist people in gaining hope and meaning when confronted with life’s difficulties as a result. Therefore, happiness and optimism, which are two important characteristics of positive mental health, are strongly related to self-compassion [34]. The explanation of such relationships can be explained by Gilbert’s regulatory model [17]. Accordingly, self-compassion is a significant predictor of well-being and health at work by activating a person’s soothing system, which aims to manage anxiety with a sense of security, trust, connection and care because the welfare-related results of self-compassion are compatible with the basis and objectives of the soothing system. Therefore, it can be assumed:

**Hypothesis 1.**  *Self-compassion has a positive and significant effect on nurses’ mental health.*

According to Key Resources Theory (KRT), self-compassion can be considered a powerful and positive internal resource of the individual [18,19], which can increase people’s resilience in difficult situations [35] and create positive psychological resources [19]. It reduces anxiety and depression [16] and acts as a preventative measure for mental health problems, and in this way, it is expected to lead to maintaining and improving the performance of people. Self-compassion also increases performance because it provides a way to decrease mental obstacles, disgusting thoughts, concerns about failure, and unpleasant feelings [36]. The effect of self-compassion on performance has been confirmed in several areas, such as sport [37], academia [38], and work [15].

Therefore, the following hypothesis can be presented:

**Hypothesis 2.**  *Self-compassion has a positive and significant effect on nurses’ job performance.*

### 2.2. Effect of Work Engagement on Mental Health and Performance

Work engagement is an important structure since it is closely related to employee well-being and job outcomes [24]. Three dimensions of work engagement are vigor, dedication and absorption [39]. Because of the nature of work engagement and its positive effects on employee well-being and life satisfaction [24], this structure is considered a critical element for organizational success and sustainability [28] and an important predictor of employee mental health status [40]. In this regard, it has been stated in studies that engaged staff can detach from their job after working hours and replenish their resources by engaging in leisure activities, which positively affects their mental health [40].

Therefore, the following hypothesis can be proposed:

**Hypothesis 3.**  *Work engagement has an effect on mental health.*

In terms of important consequences of work engagement, in some studies, work engagement has been associated with individual outcomes [41,42] and it has been cited as a key antecedent of employee output [42]. To explain this issue, we can refer to Kahn’s studies. Work engagement, according to Kahn [22], is a motivational concept that gives workers vigor and enthusiasm to perform their work tasks, and this positive energy, resulting from the appropriate motivation for better organizational performance [23], will lead to higher job performance and greater employee satisfaction [24]. On the other hand, employees with high work engagement have an emotional connection to their work because they focus longer and pay more attention to their tasks [43], and they invest their current resources in jobs and tasks, which results in higher performance [40]. Therefore, the following hypothesis can be proposed:

**Hypothesis 4.**  *Work engagement has an impact on job performance.*

### 2.3. Investigating the Impact of Self-Compassion and Work Engagement

The link between the abovementioned variables and the search for the effect of self-compassion on nurses’ work engagement is very limited and scattered.

The results of a study by Babenko et al. [44] showed that self-compassionate physicians feel more work engagement and feel less tired from their job demands; they are more satisfied with their work life. The results of another study showed a positive correlation between self-compassion and work engagement among Dutch workers [26]. In the present article, SDT and its psychological resources have been used to hypothesize the effect of self-compassion and work engagement. SDT motivates work to classify external motivation, intrinsic motivation and lack of motivation [26].

Some studies’ findings show that intrinsic motivation is critical to improving employee work engagement. Thus, what leads to the development of intrinsic motivation also strengthens work engagement because employees with intrinsic motivation maximize their work effort due to the attractiveness and challenge of the work instead of working for financial rewards [45,46]. Therefore, it can be expected that self-compassionate people have higher work engagement due to high intrinsic motivation. Then, we can hypothesize:

**Hypothesis 5.**  *Self-compassion affects work engagement.*

### 2.4. Role of Mental Health in Predicting Performance

The beneficial effects of mental health can be self-efficacy and empowerment, which are crucial preconditions for job performance and have been confirmed empirically in several studies according to social cognitive theory and cognitive motivation theories [47]. Therefore, mental health can be considered effective for job performance. Job performance in the present study was investigated as a self-perceived assessment to avoid the influence of supervisors’ perception biases.

**Hypothesis 6.**  *Mental health affects job performance.*

### 2.5. Mediating Role of Mental Health

Self-compassion is useful in promoting mental health in the workplace [10]. In human resource development research, the structure of self-compassion is known to improve employee performance through training and development with the aim of achieving mutual organizational and individual goals of employees [35].

On the other hand, since the relationship between work engagement and positive mood has been confirmed [48], in difficult and stressful situations, work engagement is expected to prevent the occurrence of psychological distress problems in the long run [49], and in this way, it maintains or improves their performance. Since mental health is also related to job performance, it can play the role of a mediating variable [26,50]. The following hypotheses can be presented as a result of this:

**Hypothesis 7.**  *Mental health has a mediating role in the effect of self-compassion on work engagement.*

**Hypothesis 8.**  *Mental health has a mediating role in the impact of job participation on job performance.*

### 2.6. Moderating Effect of Gender

Based on social role theory (SRT), there are some differences between men and women in their behaviors and expectations [19,31]. In addition, there are studies that confirmed gender differences for the self-compassion variable and observed lower levels of self-compassion among women [51]. While in some studies they did not report significant differences [52,53], they still recommend researchers and pioneers investigate gender differences in self-compassion-focused future studies and interventions.

In relation to the mental health variable, it is necessary to mention that the factors affecting the promotion of mental health cannot be considered separately from the gender of individuals, while the risks themselves are gender specific [54].

On the other hand, regarding the work engagement variable, it should be acknowledged that when the impact of work engagement on mental health outcomes and job performance is examined, the role of gender should also be considered an important variable because of gender inequality in the workplace. Many researchers have pointed to different experiences and consequences in the two gender groups due to gender role attitudes and gender stereotypes [55,56]. Therefore, it is essential to examine gender as a moderator in work engagement relationships.

**Hypothesis 9.**  *Gender has a moderating role in research model relationships.*

## 3. Material and Method

### 3.1. Sample and Procedure

To examine the research hypotheses, data were collected from intensive care unit (ICU) nurses in three coronavirus hospitals in Isfahan. According to the COVID-19 conditions and workload of nurses, after coordinating, 687 questionnaires were distributed among nurses both manually and via a Google form link, and finally, after removing redundant and incomplete questionnaires from the received questionnaires, 424 questionnaires were used as the basis for analysis. The questionnaire had two parts: demographic variables (gender and age) and questions related to the main model variables. To protect the confidentiality and privacy of individuals, the questionnaires did not need the names or signatures of the respondents.

### 3.2. Measurement of Variables and Instrument

#### 3.2.1. Mental Health

A 5-item mental health screening test was used to assess mental health [57]. Each question begins with the phrase “How much of the time, during the last month, have you……”. In this type of evaluation, mental health is measured by five single questions on a five-point Likert scale in the areas of anxiety, depression, relaxation, feeling good, emotion regulation, and behavior, ranging from rarely = 1 to always = 5. As a result, questions 1, 3, and 5 were recorded with the other three items to generate the mental health score. The function of this form of mental health assessment was confirmed by Breiwick [57].

The AUC of the MHI-5 ranged from 0.739 (anxiety disorders) to 0.892 (depression). Single MHI components were also conducted efficiently. Cronbach’s alpha for this form of health assessment was evaluated as 71.9%. In Iran, this form was used to assess the mental health of oil industry employees [58], and after confirming its validity, Cronbach’s alpha was evaluated as 0.71.

#### 3.2.2. Self-Compassion Scale (SCS-LF)

With 26 items, the Self-Compassion Scale (SCS-LF) was utilized to assess this variable [59]. The SCS-LF assesses self-compassion in six areas: self-kindness, self-judgment, common humanity feeling, isolation, awareness and over-identification. Every item is graded on a five-point Likert scale (“rarely” = 1 to “almost always” = 5). This scale assesses self-compassion by asking questions such as “my entire feeling is inferior” and “When anything bothers me, I try to keep my control of emotions”. To assess self-compassion, the scores of the questions connected to the growing component of over-identification, isolation and self-judgment must be reversed, and finally, the higher the score, the greater the self-compassion. The total score in the original version had excellent internal consistency (α = 0.92), and the CFA results supported the six subscales with values ranging from 0.75 to 0.81. In the study of Azizi et al. [60] in Iran, Cronbach’s alpha for subscales of self-compassion was evaluated as between 0.78 and 0.93. Overall, the validity and reliability coefficients of the self-compassion scale were satisfactory. Therefore, this scale is a homogeneous set with a suitable factor structure and is qualified for psychological research [60].

#### 3.2.3. Work Engagement (UWES-9)

To assess nurses’ job engagement, a reduced version of the Utrecht Work Engagement Scale (9-item version) was employed [61]. These nine components of work engagement are scored on a five-point Likert scale, with 1 signifying “strongly disagree” and 5 expressing “strongly agree”. Work engagement is measured using statements like “I feel full of energy in my work environment” and “My job inspires me. Schaufeli et al. [61] reported and validated the Cronbach’s alpha reliability of this test to be between 0.92 and 0.85. In Iran, after translation, their apparent validity and reliability were confirmed by citing experts’ opinions and Cronbach’s alpha coefficient, respectively [62]. The 9-item Utrecht Work Engagement Scale’s content validity, criterion validity, and construct validity, as well as its reliability, were confirmed (Total Alpha: 0.87). As a result, it can be used to assess nurses’ work engagement.

#### 3.2.4. Job Performance

Abramis’ Job Performance Questionnaire with 7 questions was used to measure job performance [63], and the scoring of the questionnaire was a 5-point Likert scale, with scores of 1, 2, 3, 4 and 5 considered for the options “Very Poor”, “Poor”, “Good”, “Very Good” and “Exceptionally Good”; a higher score indicates better job performance. Job performance was assessed by asking questions about two technical and social performance components, such as “fulfilling responsibilities” and “not arguing with others”. Abramis reported Cronbach’s alpha for both subscales of job performance at 0.83.

## 4. Data Analysis

In this study, we used SPSS v26 to demonstrate demographical information, descriptive statistics, and correlations between variables. Research hypotheses were also tested using AMOS24 software (version 24) and the SEM approach [64].

The SEM method is divided into two stages: the measurement and the structural model. The measurement model is concerned with measuring latent variables or composite variables, whereas the structural model investigates all conceivable dependencies using route analysis [65,66,67]. We started the analysis of the data with confirmatory factor analysis (CFA) using AMOS 24. Furthermore, the construct’s discriminant validity was assessed by comparing the square root of the average variance extracted (AVE) to the correlation between constructs. Every construct should have an AVE larger than its correlation with other constructs [68].

### 4.1. Demographics Analysis and Descriptive Statistics

To illustrate the demographic information shown in Table 1, we utilized SPSS v26. According to Table 1, 231 were females, and 193 were males, 54.5% and 45.5%, respectively. Of these, 123 (29%) of the respondents were less than 25 years old; 187 (44%) were within the age range of 25–35 years, and 114 (27%) were aged 36–45 years. As a result, the majority of respondents were less than 35 years old and the sample was gender diverse.

Table 2 shows descriptive statistics (mean, SD, Skew, and Kurt) as well as correlations between variables. According to the Pearson correlations between the variables, gender had a significant relationship with all variables, while age did not. Moreover, there were significant relationships among all the variables.

### 4.2. The Measurement Model

In this study, we used second-order CFA with Brown’s approach [69]. The result of second-order CFA shows the standardized factor loading and T_value for each item were greater than 0.6 and 1.96, respectively. In addition, for each construct, AVE > 0.5 and CR > 0.7 are shown and can confirm convergent validity and reliability [70,71,72,73,74,75,76]. Therefore, according to Table 3, the measurement of the model was internally consistent.

### 4.3. Formatting of Mathematical Components

Table 4 compares the correlation coefficients between constructs and the √AVE on the Table’s diagonal. The values of the √AVE were greater than the correlation coefficients between the variables, as seen in Table 4; this demonstrates that the constructs’ discriminant validity was confirmed.

Furthermore, we examined discriminant validity with the monotrait–heteromethod (HTMT) 0.85 [77].

According to Table 5, the ratio of HTMT correlations was less than the 0.85 cutoff values; therefore, the constructs had discriminant validity.

### 4.4. The Structural Model

**Figure 1:**
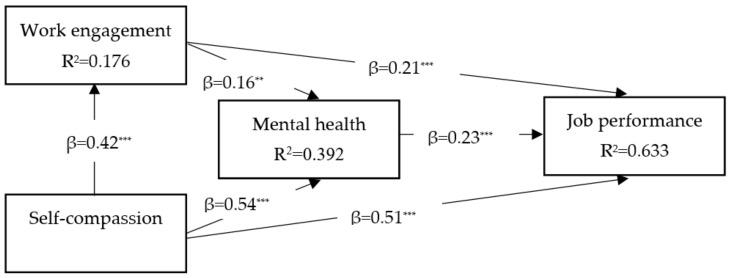
Path coefficients and significance level. *** *p* < 0.001, ** *p* < 0.01.

The structural equation model findings displayed the values CMIN/DF = 0.964, CFI = 1.000, SRMR = 0.019, RMSEA = 0.000 and PClose = 1.000. These outcomes show model fit indicators were within the standard acceptable range [78]. Furthermore, Figure 1 represents the path coefficients related to the hypothesis.

The results demonstrate that self-compassion had a significant effect on work engagement (β = 0.42, *p* < 0.001), mental health (β = 0.54, *p* < 0.001), and job performance (β = 0.51, *p* < 0.001). Furthermore, work engagement had a significant influence on mental health (β = 0.16, *p* < 0.01) as well as job performance (β = 0.21, *p* < 0.001). Finally, the impact of mental health on job performance was statistically significant (β = 0.23, *p* < 0.001).

Table 6 examines the indirect consequences as well as the direct effects. As is shown, the indirect effects of self-compassion and work engagement on job performance via mental health were confirmed. As a result, mental health is a mediator for these relationships.

### 4.5. Moderation Impacts

Independent *t*-tests were conducted to specify whether the mean of variables differed in the two gender groups. Results indicated that the mean of all variables was higher in the male group than in women, and all of these differences were significant. In addition, the mean value of mental health (mean = 2.44) in the women’s group was lower than the average of the Likert scale (average = 3), which means that the level of mental health of women nurses was not in good condition.

The multi-group test used the intensity of the path coefficient to assess the moderating effect of gender differences. Two groups for males and females were initially created, and then the multi-group test was executed through AMOS24. According to the results of the multi-group test in Table 7 and Table 8, gender moderates the relationship between self-compassion and work engagement, work engagement and job performance, and self-compassion and job performance. In all these relationships, the path coefficients for males were higher than those for females. The insignificance of the remaining correlations indicates that gender was not a moderator in these relationships.

## 5. Discussion

The study aimed to examine the role of mental health as a mediator and gender as a moderator in the influence of self-compassion and work engagement on the job performance of ICU nurses in hospitals in Isfahan, Iran. This study investigated the path coefficients and their significance using AMOS24 software (version 24). Findings can expand the role of mental health in the impact of job performance antecedents (self-compassion and job conflict) in stressful situations such as pandemics in the occupational group of nurses. This research advances our understanding of the elements that influence mental health and occupational success as well as, by providing empirical evidence, emphasizing the need for interventions to promote these elements among nurses. The results of the model implemented by AMOS24 and the validity of the research hypotheses are as follows.

According to research theories, nurses’ self-compassion improves their mental health, work engagement and job effectiveness. Data analysis validated hypotheses 1, 2 and 5, revealing that self-compassion in nurses had a favorable impact on mental health (β = 0.54, *p* < 0.001), work engagement (β = 0.42, *p* < 0.001) and job performance (β = 0.51, *p* < 0.001). It is positively effective and meaningful and is consistent with previous studies [10,15,26,29,44]. These results showed that nurses who were more self-compassionate had higher mental health status. Self-compassion as a positive characteristic and a valuable positive source of coping in times of intensified stress, such as the COVID-19 condition, could have a positive effect on mental health and protect nurses’ mental health. In addition, self-compassion could have a positive effect on job performance by overcoming mental barriers and reducing the fear of failure because self-compassion acts by thinking about stressful situations in a way that reinforces adaptation. People who have more self-compassion are unlikely to use avoidant coping strategies by accepting responsibility for negative events. In fact, instead of running away and creating distractions, they face the situation and, by accepting it, try to provide the best reaction in those situations [79]. According to the meaning of self-compassion, people who are more compassionate towards themselves have more kindness towards themselves in the time of COVID-19, see that it is a global problem that has caused all nurses with more workload and stress, do not consider it a personal problem, and they are less prone to rumination and anxiety, which leads to the prevention of mental health problems. They also, by accepting problems and situations and not being afraid of failing to perform challenging tasks, show better job performance than people who have less self-compassion. Examining the effect of self-compassion on work engagement also showed that self-compassion can promote work engagement. This can be in line with SDT theory and psychological sources of motivation. Self-compassion, as it can strengthen intrinsic motivation and is more associated with a positive cognitive reconstruction coping strategy, can result in a lack of focus on negative emotions, optimism, happiness and a positive affect [18,19,79]. Under COVID-19 conditions, when working is stressful, these positive emotions and motivation, as the main preconditions for work engagement, are able to increase work engagement in people who have more self-compassion.

Work engagement was found to have a favorable influence on nurses’ mental health and job performance, which is consistent with Hypotheses 3 and 4 and earlier research [24,26,80]. There are two views on the impact of work engagement on mental health; on the one hand, some researchers have confirmed work engagement as a predictor of mental health [40] and on the other hand, some claim the dark effects of work engagement on mental health and job performance. In the study by Shimazu et al. [49], they confirmed only the dark aspect of work engagement in the short term on mental health, and no negative effects on mental health and performance were witnessed. The present study also showed that nurses who have more engagement at work are more likely to put forth more effort, which leads to increased performance, while at the same time, improving their mental health. Thus, work engagement through the motivational mechanism is able to positively predict mental health and job performance.

The results of the structural equation model and its significance for Hypothesis 6 also showed that mental health has a positive and significant effect on nurses’ job performance (β = 0.23, *p* < 0.001). This is consistent with previous research [9,26,81]. There are also meta-analytic studies that have confirmed a negative association between job performance and adverse mental health outcomes [82]. This could be explained considering that mental disorders that manifest as depression and other emotional disorders lead to reduced productivity and job performance due to a severe reduction in work ability [83]. Therefore, nurses who have better mental health also show better job performance.

In addition to the direct effect of the variables of self-compassion and work engagement on nurses’ job performance, the study of the mediating role of mental health in relation to hypotheses 7 and 8 showed that this variable can to some extent mediate the relationship of self-compassion and work engagement on job performance and finally these variables were able to predict 63% changes in job performance through direct and indirect paths (R^2^ = 63%). Confirmation of the mediating effect means that the impact of work engagement and self-compassion on job performance can be justified by considering the impact they have on mental health. Self-compassion overcomes mental barriers due to self-kindness and acceptance of problems, and work engagement reduces depression and anxiety by motivating and energizing, thereby maintaining the concentration of people in their job and preventing a reduction in performance, as job performance could reduce when people have mental health problems. Results of the gender moderation role in relation to hypothesis 9 revealed that the effects of self-compassion and work engagement on mental health, and the mental health influence on job performance, were effective and significant in both gender categories, with insignificant differences observed between the two groups (Z < 1.96). This result is consistent with some previous studies. The role of gender moderation in self-compassion relationships with its consequences, such as emotional well-being among the adolescent population and health behaviors in the general population, has been investigated in studies that have not been confirmed [53,84].

This result shows that apart from gender, the effect of self-compassion and work engagement has been able to positively and significantly affect the mental health of nurses, and their mental health status has a positive and significant effect on their job performance. Therefore, strategies to strengthen these two predictor variables will be effective for both gender groups to promote mental health. The direct effects of self-compassion and work engagement on job performance, as well as the effect of self-compassion on work engagement, were significantly different (Z > 1.96) in the two sex classes (impact coefficients were higher in men than women), and the direct work engagement effect on job performance was not significant in women (β = 0.058, *p* > 0.05).

The regression coefficients among men indicate that self-compassion and work engagement in stressful conditions have led to improved performance and work engagement, while for women, they have only led to the promotion of mental health. It may be due to women’s lower perceptions of their performance and success in return for their efforts compared to men. Men appear more optimistic, active and responsive to problems than women because women are emotional and have negative expectations about problems [85,86], which can lead to different behavioral outcomes between the two categories. In addition, some women perceive a “glass ceiling” in an organization, and it has been empirically shown that this negatively affects women’s perceptions of their job success [55]. Although women are more likely than men to participate in service occupations such as nursing, due to the unfair conditions mentioned in the workplace and different social and family roles between men and women [87], gender can affect work engagement and relationships. Studies have also shown the effect of gender on work engagement among nurses [78] and IT staff in India [88], and have examined and confirmed the role of gender moderation in predictors of work engagement [89]. The result of this section can also be due to the presence of a mental health variable as a mediator, which may play the role of a complete mediator in the group of women. Therefore, in the field of formulating and applying strategies to promote job performance in female nurses, other variables that can be the main predictors of job performance and mental health in this gender group should be identified and examined, and also mediation role of mental health should be examined separately in two group of gender.

As can be seen in the mean of the variables (Table 2), the mental health of the ICU nurses who participated in the study (mean: 2.79) is not in good condition, which determines the urgency of the investigation and action in this field. The study by Zakeri et al. [8] showed that the incidence of GAD (generalized anxiety disorder) and PTSD (post-traumatic stress disorder) were high among workers in the healthcare field in Iran. In the research conducted in other countries, the results indicate a low level of mental health among nurses [90]. In addition, results of the difference in mental health status in the two gender groups showed that women’s mental health is poorer than men’s, as shown proven in China and other countries [90,91]. The theoretical literature states that the difference in the rate of depression between men and women can be due to the use of different coping styles in stressful situations as well as differences in the level of perceived social support. Women are more likely to respond to stressful situations with more anxiety and rumination, which is strongly associated with depression [92]. Research by Thapar et al. [93] also found that adolescent girls experience twice as much anxiety as their male peers.

## 6. Implications

Despite the limitations of this study, it provided a fundamental insight into the effects and relationships of self-compassion, work engagement and mental health on nurses’ job performance during COVID-19 conditions. Strengthening self-compassion for nurses is useful and important given their working conditions, especially in situations where their work stress could increase due to pandemic conditions, war conditions, or natural disasters such as floods and earthquakes. The results of nurses’ inability to manage their stress, as experience and studies in the last two years have shown, have worsened their physical and mental problems (heart attack, depression), decreased job performance, increased frequent mistakes in performing tasks, leaving work and suicide among this stratum. Due to the occupational nature of nurses, it is necessary to consider the factors that can improve their mental health and ability to overcome stress in order to maintain their physical and mental health, in addition to improving their job performance. Therefore, based on the insight presented by this study in Hypotheses 1 and 2, self-compassion has led to better mental health and job performance. In terms of the hypothesis, the positive effect of self-compassion on work engagement was confirmed. Based on these findings, psychologists should maintain and promote nurses’ mental health and job performance, and should pay more attention to their self-compassion. Therefore, in critical COVID-19 pandemic situations in hospitals, self-compassion can act as a potential shield in a hospital when nurses experience emotional distress in caring for patients because compassion is a useful response of a person to the sufferings and problems of others [7]. Some people are more self-compassionate than others, either naturally or for other reasons [84]. Therefore, this feature can be used as one of the selection criteria for nurses to work in the ICU in hospitals. In addition, providing appropriate treatment plans, such as a mindful self-compassion program, can be useful to strengthen nurses’ self-compassion [33]. According to the findings in relation to Hypotheses 3 and 4, human resource managers in hospitals should consider strengthening the work engagement of nurses as a strategy to strengthen mental health and performance [29,42]. They should use different and new methods such as mindfulness interventions, job crafting, using appropriate leadership styles and appropriate nursing workload adjustment to increase nurses’ work engagement.

This study makes an essential contribution by looking into the impact of mental health in predicting job success through self-compassion and work engagement (Hypotheses 6, 7 and 8). Zakeri et al. [8] emphasize the need to provide counseling programs and training in psychological skills to employees to strengthen mental health.

As a result, paying attention to the nurse’s mental health throughout the COVID-19 pandemic should be one of the hospital personnel department’s top objectives. Relevant officials should prevent the occurrence of mental health problems among nurses in order to maintain nurses’ job performance by implementing intervention programs and referring nurses with symptoms to psychologists to treat and receive necessary solutions. In order to improve the mental health of nurses, it is necessary to implement various policies in the workplace and society, such as considering social benefits, increasing salaries and appreciating the community of nurses.

Furthermore, the results of the gender moderating function regarding Hypothesis 9 revealed that the effects of self-compassion and work engagement on nurses’ job performance were greater in males than females, but no significant influence was found in other linkages. Because of the lack of significant differences between the two groups in some relationships (the effects of self-compassion and work engagement on mental health, and the mental health impact on job performance), as well as lower average variables in women, which could be attributed to Asian culture and traditional gender roles [51], it seems to be especially important to help women learn how to empathize with themselves instead of criticizing themselves to increase their mental well-being and how to protect their mental health by promoting motivation and energy to do work.

According to the difference in averages in the two groups, women’s mental health is more at risk than men’s. Therefore mental health programs should pay more attention to women and teach them coping strategies in the face of stress, and women’s mental health status should be monitored carefully, especially during the pandemic. To provide more adaptive strategies for this gender class, more in-depth and comprehensive studies should be carried out. In terms of work engagement, women are less motivated than men to have more engagement at work, which can help HR managers in hospitals to eliminate gender discrimination in promotion, rewards and career paths in order to motivate female nurses to increase their work engagement at work.

## 7. Limitations and Future Direction

The most important limitation of this study was that few hospitals agreed to participate in this study because of the COVID-19 conditions. In addition, access to nurses to distribute and collect questionnaires was very difficult, and a number of questionnaires were incomplete. In this study, data was collected through a self-reported questionnaire. This may lead to common methodological biases. For example, nurses’ job performance was measured in the form of a self-reported questionnaire because of supervisors’ high workload that led to not participating in this study. Therefore, in future research, nurses’ job performance can be measured by supervisors based on their annual job performance or other methods. This study also does not consider the role of culture in the study of relationships since the main variables of research, such as self-compassion and mental health, can be influenced by culture [94]. It is suggested that future research, while examining these relationships in other countries, should study these relationships through a comparative study in countries with different cultures. The next limitation is the lack of studies about the role of mental health mediation in performance prediction, so while innovating and expanding the theoretical literature of this research in this section, the comparison of the results of this hypothesis was limited. In the present study, only the effects of self-compassion and work engagement on mental health and job performance were investigated. In future research, the effects of various positive coping strategies as a mental health protector can be examined. Also, the mediating role of mental health in separate gender groups can provide researchers with a clearer understanding of relationships. In terms of gender’s moderating role, it can be stated that, in addition to gender differences, differences in personality qualities such as neurotic and conscience might influence the impact of self-compassion on mental health, so the role of these traits as a moderator variable can be considered in future studies by researchers. Researchers can also examine the interaction between age and gender as a moderator variable at the same time because if gender differences are found in specific groups, a special emphasis can be placed on maximizing the positive consequences of self-compassion, work engagement, and mental health in specific populations. The sample in the present study was ICU nurses, and the relationship between the model and hypotheses was examined only in this group. It could also be carried out in non-nursing jobs and in occupational groups with higher social rank in the future.

## 8. Conclusions

This study looked at the links between self-compassion, work engagement and job performance, both directly and indirectly via mental health. In addition, the model was compared between the two gender groups. Results showed that during the COVID-19 pandemic, in the entire statistical sample, self-compassion and work engagement directly on job performance are effective, and mental health also plays a mediating role. The effects of self-compassion and work engagement on mental health were not statistically different across men and women. However, the Impact coefficients of self-compassion and work engagement on job performance were lower in women. These findings give theoretical and empirical support for psychological intervention to improve ICU nurses’ job performance and mental health. The research results support the view that in the nursing community, people who have higher self-compassion and work engagement can be employed in the ICU. Interventions can also be used to improve these two variables. On the other hand, since mental health was confirmed as a predictor of job performance and mediation, it is important to pay attention to the mental health status of nurses at the time of selection and employment, especially in crucial situations, and specific emphasis should be placed on implementing ways to improve mental health and prevent mental health disorders. Furthermore, given the favorable outcomes of self-compassion, work engagement and mental health, it is preferable to make efforts to increase these variables for all nurses, regardless of gender.

## Figures and Tables

**Table 1:** Demographic Information of the Sample.

Category	Property	Frequency	Percentage
**Gender**	Female	231	54.5%
	Male	193	45.5%
**Age**	>25	123	29%
	25–35	187	44%
	34–45	114	27%

**Table 2:** Results of descriptive statistics, skewness, kurtosis and Pearson correlations.

	**Mean**	**S.D**	**Skew**	**Kurt**	**1**	**2**	**3**	**4**
**1. Woeng**	3.895	0.785	−0.520	−0.174	1			
**2. Secom**	3.475	0.704	−0.468	−0.407	0.374 **	1		
**3. Mehel**	2.793	0.934	0.042	−0.654	0.343 **	0.553 **	1	
**4. Jobpe**	3.823	0.777	−0.405	−0.432	0.437 **	0.639 **	0.535 **	1

Woeng = work engagement, Secom = self-compassion, Mehel = mental health, Jobpe = job performance. ** *p* < 0.01.

**Table 3:** Results for reliability and convergent validity in CFA.

Constructs/Items	Standardized Factor Loading in CFA	T_Value in CFA	Composite Reliability (CR)	Average Variance Extracted (AVE)
**Work Engagement**			0.924	0.803
**Vigo**	0.935	15.339	0.869	0.689
**Vigo1**	0.78	18.494		
**Vigo2**	0.865	21.404		
**Vigo3**	0.844			
**Dedi**	0.869	14.419	0.882	0.713
**Dedi1**	0.866	20.521		
**Dedi2**	0.846	19.948		
**Dedi3**	0.821			
**Abso**	0.883		0.881	0.711
**Abso1**	0.836	19.908		
**Abso2**	0.865	20.813		
**Abso3**	0.829			
**Self-Compassion**			0.941	0.725
**Seki**	0.779	14.218	0.922	0.705
**Seki1**	0.883	26.47		
**Seki2**	0.727	18.44		
**Seki3**	0.821	22.835		
**Seki4**	0.86	25.015		
**Seki5**	0.895			
**Seju**	0.845	13.663	0.888	0.613
**Seju1**	0.801	17.598		
**Seju2**	0.77	16.763		
**Seju3**	0.792	17.334		
**Seju4**	0.768	16.698		
**Seju5**	0.784			
**Cohu**	0.884	14.186	0.885	0.661
**Cohu1**	0.875	19.994		
**Cohu2**	0.886	20.299		
**Cohu3**	0.689	14.859		
**Cohu4**	0.784			
**Isol**	0.843	13.736	0.861	0.608
**Isol1**	0.753	16.364		
**Isol2**	0.835	18.502		
**Isol3**	0.728	15.683		
**Isol4**	0.799			
**Mifu**	0.867	15.384	0.881	0.652
**Mifu1**	0.67	15.787		
**Mifu2**	0.803	20.868		
**Mifu3**	0.857	23.368		
**Mifu4**	0.884			
**Ovin**	0.886		0.878	0.642
**Ovin1**	0.812	17.991		
**Ovin2**	0.827	18.415		
**Ovin3**	0.777	17.033		
**Ovin4**	0.788			
**Mental health**			0.909	0.668
**Mehe1**	0.82	18.477		
**Mehe2**	0.806	18.078		
**Mehe3**	0.855	19.482		
**Mehe4**	0.812	18.257		
**Mehe5**	0.79			
**Job performance**			0.874	0.776
**Soci**	0.881		0.883	0.716
**Soci1**	0.833	20.618		
**Soci2**	0.848	21.117		
**Soci3**	0.857			
**Tech**	0.881	13.788	0.878	0.644
**Tech1**	0.847	20.088		
**Tech2**	0.709	15.839		
**Tech3**	0.821	19.274		
**Tech4**	0.825			
**Model Fit: x^2^/df = 1.098, CFI = 0.993, SRMR = 0.03, RMSEA = 0.015, PClose = 1.000**				

Vigo = vigor, Dedi = dedication, Abso = absorption, Seki = self-kindness, Seju = self-judgment, Cohu = common humanity, Isol = isolation, Mifu = mind fullness, Ovin = over indemnification, Soci = social, Tech = technical.

**Table 4:** Correlation matrices and the √AVE.

	1	2	3	4
**1. Mental health**	0.817			
**2. Work engagement**	0.385 ***	0.896		
**3. Self-compassion**	0.611 ***	0.424 ***	0.852	
**4. Job performance**	0.625 ***	0.516 ***	0.738 ***	0.881

*** *p* < 0.001.

**Table 5:** Discriminant validity (HTMT).

	1	2	3	4
**1. Mental health**				
**2. Work engagement**	0.373			
**3. Self-compassion**	0.592	0.397		
**4. Job performance**	0.589	0.475	0.683	

**Table 6:** Direct and Indirect Effects.

Direct Path	Unstandardized Estimate	Standardized Estimate	S.E	C.R	*p*-Value
**Self-compassion -> Work engagement**	0.413	0.42	0.053	7.81	***
**Work engagement -> Mental health**	0.206	0.157	0.066	3.11	0.002
**Self-compassion -> Mental health**	0.698	0.543	0.0.71	9.88	***
**Work engagement -> Job performance**	0.209	0.215	0.047	4.44	***
**Self-compassion -> Job performance**	0.488	0.51	0.057	8.49	***
**Mental health -> Job performance**	0.172	0.231	0.041	4.14	***
**Indirect Path**	**Unstandardized Estimate**	**Standardized Estimate**	**Lower**	**Upper**	***p*-Value**
**Self-compassion -> Mental health -> Job performance**	0.12	0.125 **	0.071	0.18	0.001
**Work engagement -> Mental health -> Job performance**	0.035	0.036 **	0.014	0.064	0.006

*** *p* < 0.001. ** *p* < 0.010.

**Table 7:** Comparisons of male and female nurses on variables.

Variables	Mean (SD)	Mean (SD)	*t*-Test	*p*
	Male	Female		
**1. Woeng**	4.18 (0.73)	3.65 (0.74)	−7.40 **	0.000
**2. Secom**	3.90 (0.57)	3.11 (0.58)	−14.06 **	0.000
**3. Mehel**	3.20 (0.84)	2.44 (0.86)	−9.09 **	0.000
**4. Jobpe**	4.28 (0.67)	3.44 (0.64)	−13.12 **	0.000

**Woeng =** work engagement, **Secom =** self-compassion, **Mehel =** mental health, **Jobpe =** job performance. ** *p* < 0.01.

**Table 8:** Multi-group analysis with gender as a moderator.

Path			Female		Male		z-Score
			Estimate	*p*	Estimate	*p*	
**Work engagement**	**<-**	**Self-compassion**	0.172	0.038	0.459	0.000	2.399 **
**Mental health**	**<-**	**Work engagement**	0.144	0.036	0.219	0.006	0.716
**Mental health**	**<-**	**Self-compassion**	0.648	0.000	0.480	0.000	−1.257
**Job performance**	**<-**	**Mental health**	0.173	0.000	0.182	0.000	0.136
**Job performance**	**<-**	**Work engagement**	0.058	0.285	0.248	0.000	2.792 ***
**Job performance**	**<-**	**Self-compassion**	0.155	0.042	0.650	0.000	5.306 ***

** *p* < 0.05, *** *p* < 0.01.

## Data Availability

Not applicable.
